# Analyses of *Gnai3*-iresGFP reporter mice reveal unknown Gα_i3_ expression sites

**DOI:** 10.1038/s41598-021-93591-0

**Published:** 2021-07-12

**Authors:** Veronika Leiss, Ellen Reisinger, Annika Speidel, Sandra Beer-Hammer, Bernd Nürnberg

**Affiliations:** 1 Department of Pharmacology, Experimental Therapy and Toxicology, Institute of Experimental and Clinical Pharmacology and Pharmacogenomics, and ICePhA Mouse Clinic, University of Tübingen, Wilhelmstraße 56, 72074 Tübingen, Germany; 2 Department of Otolaryngology-Head and Neck Surgery, Gene Therapy for Hearing Impairment Group, University of Tübingen, Medical Center, Elfriede-Aulhorn-Straße 5, 72076 Tübingen, Germany

**Keywords:** Cell biology, Molecular biology

## Abstract

Inhibitory G proteins (G_i_ proteins) are highly homologous but play distinct biological roles. However, their isoform-specific detection remains challenging. To facilitate the analysis of Gα_i3_ expression, we generated a *Gnai3﻿﻿-* ﻿iresGFP reporter mouse line. An internal ribosomal entry site (IRES) was inserted behind the stop-codon of the *Gnai3* gene to initiate simultaneous translation of the GFP cDNA together with Gα_i3_. The expression of GFP was confirmed in spleen and thymus tissue by immunoblot analysis. Importantly, the GFP knock-in (ki) did not alter Gα_i3_ expression levels in all organs tested including spleen and thymus compared to wild-type littermates. Flow cytometry of thymocytes, splenic and blood cell suspensions revealed significantly higher GFP fluorescence intensities in homozygous ki/ki animals compared to heterozygous mice (+/ki). Using cell-type specific surface markers GFP fluorescence was assigned to B cells, T cells, macrophages and granulocytes from both splenic and blood cells and additionally blood-derived platelets. Moreover, immunofluorescent staining of the inner ear from knock-in mice unraveled GFP expression in sensory and non-sensory cell types, with highest levels in Deiter’s cells and in the first row of Hensen’s cells in the organ of Corti, indicating a novel site for Gα_i3_ expression. In summary, the *Gnai3-﻿﻿* iresGFP reporter mouse represents an ideal tool for precise analyses of Gα_i3_ expression patterns and sites.

## Introduction

G protein-coupled receptors (GPCRs) and their G proteins are key players in cellular signalling and therefore represent important drug targets^1–4^. Upon binding of an extracellular agonist to its cognate GPCR, the G protein transduces the signal from the activated GPCR to an intracellular second messenger generating system. Malfunctions of G proteins or GPCRs are known to underlie a series of major health burdens, such as metabolic disorders or cancer. Understanding the cell-type-specific functions and underlying mechanisms of G protein-dependent signalling is therefore crucial for the development of new drugs. The heterotrimeric G proteins, composed of an α-, β- and γ-subunit, are classified into four families, due to their sequence homologies and functions of the α-subunits, each controlling a distinct profile of effectors^5^. The three Gα_i_ isoforms (Gα_i1_, Gα_i2_, Gα_i3_) of the inhibitory G_i_/G_o_ family are closely related, with a sequence homology of 88–94%, with Gα_i1_ and Gα_i3_ exhibiting the highest homology (94%) followed by Gα_i3_ and Gα_i2_^6^.

G_i_ proteins are ubiquitously expressed, with distinct and partially overlapping expression patterns of the individual isoforms. Gα_i1_ is mainly expressed in neuronal tissues and represents, together with Gα_o_, the predominant type of Gα_i_/Gα_o_ proteins within neuronal structures^7^. Gα_i2_ is the most prevalent and ubiquitously expressed isoform, and we and others have demonstrated its involvement in many physiological and pathophysiological functions^8–14^. In contrast to Gα_i2_, Gα_i3_ is found in lower levels, and it is mainly detectable in non-neuronal organs and tissues and only barely in the brain^7,11,12,15–18^.

Due to the high homology and partially overlapping expression patterns of the Gα_i_ isoforms, different transgenic mouse models with specific gene knock-out or over-expression as well as loss-of-function mutations have been extremely valuable to decipher individual isoform functions^19–21^. Global Gα_i2_-deficiency induces various phenotypes, the most obvious affecting the immune-, metabolic- and cardiovascular system^8,14,22–25^. Of note, double knock-out of Gα_i2_ and Gα_i3_ results in intrauterine death^16^. In addition, expression analyses of Gα_i3_ in Gα_i2_-deficient mice often shows an upregulation of Gα_i3_, suggesting that Gα_i3_ compensates for the lack of Gα_i2_ to some extent^8,11,12,18,26^. Despite such a partial redundancy in function, the mouse models display distinct phenotypes indicating isoform-specific biological roles. Particularly, for the minor expressed Gα_i3_ isoform, these isoform-specific functions become more and more evident: (1), Gα_i3_ but not Gα_i2_ mediates insulin-induced control of autophagy in murine livers^16^, (2) in anatomical studies of the mouse skeleton, lack of Gα_i3_ resulted in fusions of ribs and lumbar vertebrae during embryonic development^20^, (3) in addition, reduced cardiac infarction in myocardial ischemia reperfusion injury was observed in Gα_i3_ knock-out mice^18^, (4) in the inner ear, Gα_i3_ deficiency impaired migration of the kinocilium at the surface of cochlear hair cells, which resulted in disarranged hair bundle orientation and shape^27^. This led to elevated hearing thresholds for high-frequency sound^15,27–29^. Due to the low expression levels of Gα_i3_ per se and the high sequence homology of the Gα_i_ isoforms, it is difficult to unambiguously detect Gα_i3_. Although isoform-specific antibodies have been generated, sophisticated and time-consuming SDS-PAGE techniques such as gradient-, urea-supplemented or 2D-gels for immunoblot analysis have to be performed to sufficiently separate the Gα_i_ isoforms and to reliably identify Gα_i3_^30,31^. Additionally, there is an ongoing discussion whether the expression of Gα_i3_ is indeed restricted to non-neuronal compartments, or whether it is also expressed in neuronal structures. Thus, it remains a continuing challenge to specifically identify Gα_i3_ in tissue homogenates, flow cytometry and immunostainings. To circumvent interference of a GFP-Gα_i3_ fusion protein with proper localization and/or function of Gα_i3_, we generated an IRES-driven GFP-reporter mouse model, termed *Gnai3﻿* -iresGFP reporter mouse, and aimed to use *Gnai3*-driven GFP expression as a convenient and precise identification tool to analyse and conclude on the sites of Gα_i3_ expression.

## Results

In this study, we investigated whether the GFP expression coupled to *Gnai3* transcription can be visualized and used as a surrogate parameter for expression and quantification of Gα_i3_.

### Generation of Gnai3-iresGFP reporter mice

**Figure 1:**
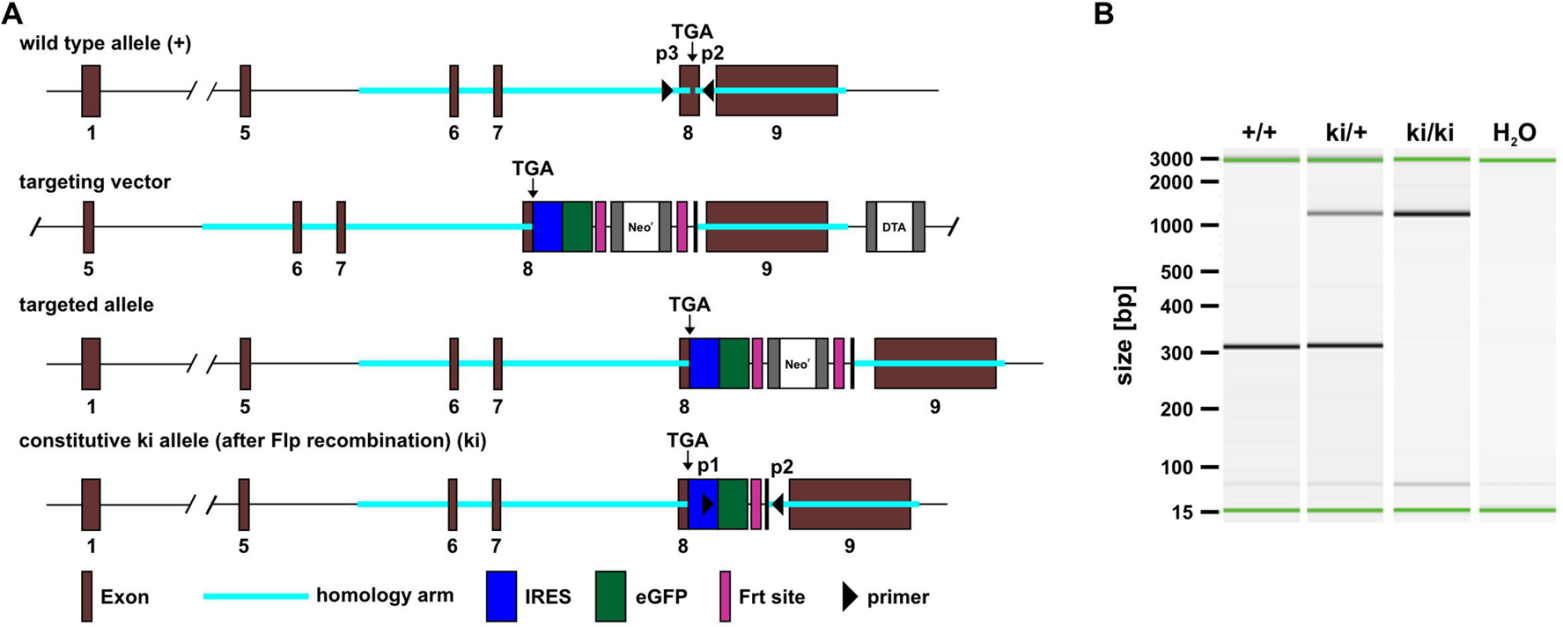
Generation and characterization of *Gnai3*-iresGFP reporter mice. (**A**) Gene targeting strategy to generate the *Gnai3*-iresGFP mouse line. In wild-type animals the *Gnai3* gene is localized on chromosome 3 and consists of nine exons. The stop codon for *Gnai3* transcription is localized in exon 8. The targeting vector contained exon 5 to exon 9, two homology arms, an internal ribosomal entry site (IRES) directly inserted behind the TGA stop codon for Gα_i3_ in exon 8, the cDNA coding for GFP, a Neo^r^ cassette flanked by two Frt sites and a diphteria toxin A (DTA) cassette outside the second homology arm. For positive selection the neomycin resistance (Neo^r^) was used, whereas the expression of DTA guaranteed the cell death upon random integration. Following homologous recombination, the targeting vector has been integrated at the respective position in the targeted allele. Finally, after Flp recombination, a constitutive ki allele had been generated. (**B**) Representative PCR analysis from ear biopsies of wild-type (+/+), heterozygous (+/ki) and homozygous knock-in (ki/ki) mice. The 300 bp fragment represents the wild-type, amplification of the knock-in fragment results in a 1200 bp product. H_2_O was used as negative control.

Aiming to detect *Gnai3* transcripts via expression of GFP, we integrated an IRES-GFP expression cassette behind the stop codon of the *Gnai3* locus in the mouse genome to generate the *Gnai3* -iresGFP reporter mouse line (Fig. 1A). Thus, Gα_i3_ and GFP are transcribed in a bicistronic mRNA but subsequently translated into two independent proteins rather than as a fusion protein. This shall ensure unaffected Gα_i3_ transcription and function and simultaneous GFP expression as a reporter protein. All animals were fertile and showed normal behavior and no obvious phenotype.

PCR analysis on ear biopsies confirmed the presence of two knock-in alleles in homozygous (ki/ki) animals, indicated by the 1200 bp sized PCR product (Fig. 1B). The wild-type (+) allele was verified with the 300 bp sized PCR product. Consistent with this, in biopsies of heterozygous (+/ki) animals, both the 300 bp wild-type and the 1200 bp knock-in bands were detectable. Thus, the IRES-GFP construct has been successfully inserted into the *Gnai3* locus.

### GFP expression is detectable in spleen and thymus

**Figure 2:**
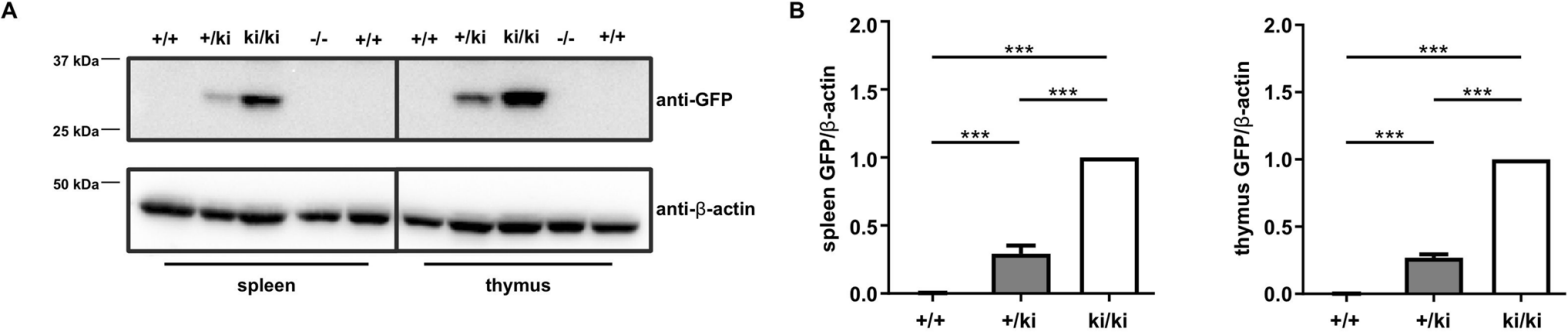
GFP expression in *Gnai3*-iresGFP reporter mice. (**A**) Representative immunoblots of GFP expression in spleen and thymus derived from wild-type, heterozygous and homozygous *Gnai3*-iresGFP mice. 20 µg protein lysate were loaded in each lane. Equal loading was confirmed by β-actin detection. (**B**) Statistical analysis of GFP expression in spleen (left) and thymus (right) of *Gnai3*-iresGFP mice. The intensities of the GFP bands were normalized to the relative intensity of the loading control β-actin (n = 5 per genotype). Data are represented as mean ± SD and were analyzed using multiple comparison one-way ANOVA followed by post-hoc comparison with Bonferroni’s multiple comparison. Uncropped blots are shown in Supplemental Figure S2.

Next, we examined for expression of GFP proteins. Immunoblot analysis with GFP-antibody staining of spleen and thymus derived from wild-type, heterozygous and homozygous knock-in mice revealed the GFP signal only in mice carrying the knock-in allele(s) (Fig. 2A). In wild-type and *Gnai3*-deficient spleen and thymus homogenates, GFP expression was not detectable (Fig. 2A). Statistical analysis confirmed the presence of GFP in +/ki and ki/ki tissues (Fig. 2B). Of note, GFP expression levels in homozygous knock-in animals were significantly higher compared to heterozygous mice. This indicates that both *Gnai3* alleles are transcribed for Gα_i3_ protein expression.

### Knock-in of GFP does not alter Gα_i3_ protein expression

**Figure 3:**
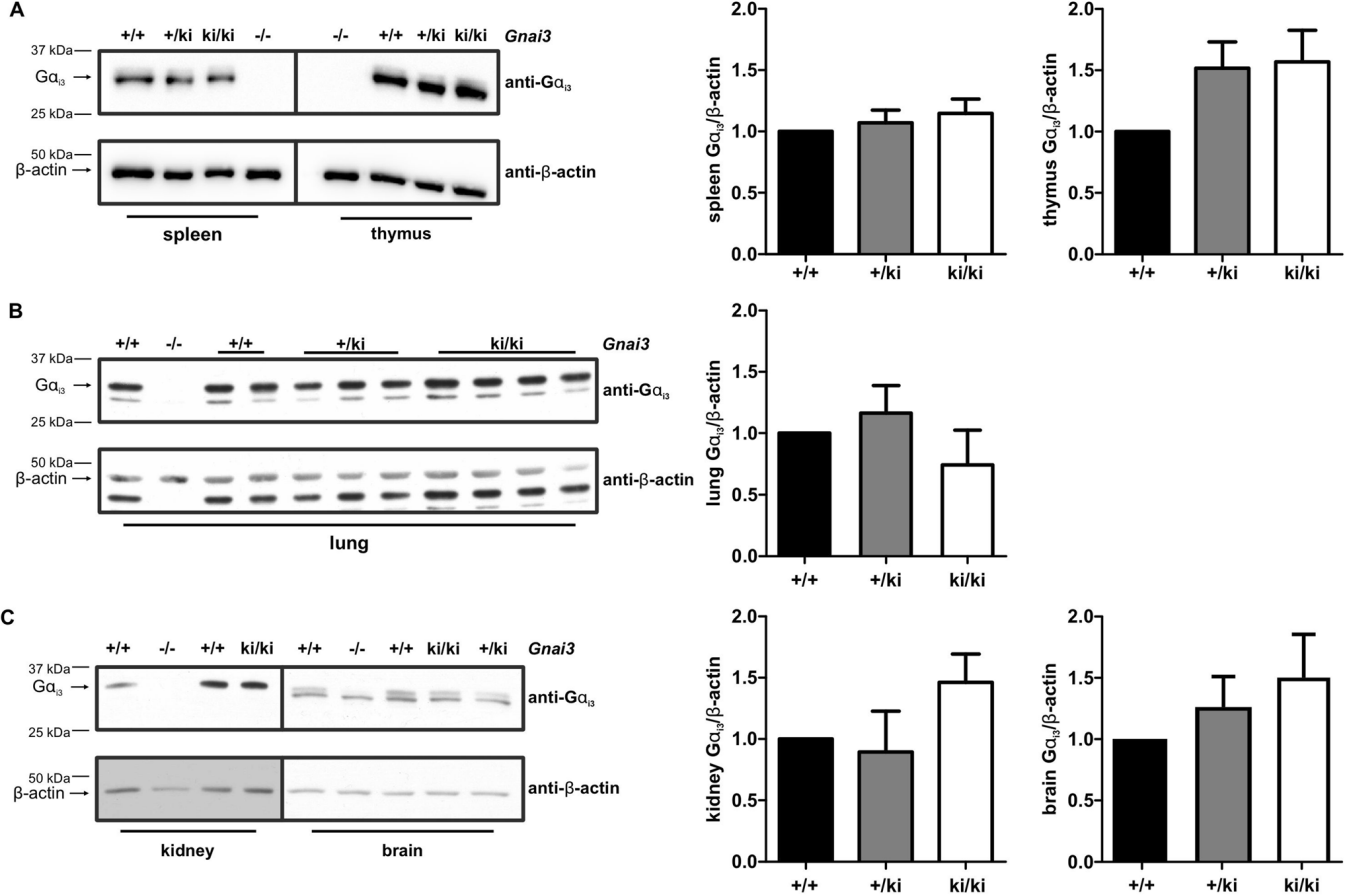
Gα_i3_ expression in tissues of *Gnai3*-iresGFP reporter mice. (**A**) Representative immunoblot analyses showing Gα_i3_ expression in spleen and thymus homogenates and statistical analysis of Gα_i3_ expression levels in spleen (n = 14 for each genotype) and thymus (n = 4 for each genotype) of wt, +/ki and ki/ki animals. (**B**) Representative immunoblot and statistical analysis of Gα_i3_ expression in lung (n = 6 for each genotype) homogenates of wild-type and *Gnai3*-iresGFP reporter mice. (**C**) Representative immunoblot and statistical analyses of Gα_i3_ expression in kidney (n = 5 for each genotype) and brain (upper band; n = 4 per genotype) of homozygous and heterozygous *Gnai3*-iresGFP mice compared to littermate controls. The Gα_i3_-specific antibody is directed against the last 21 amino acids of the C-terminal sequence of the Gα_i3_ protein. To verify antibody specificity protein homogenates from global *Gnai3* (−/−) deficient mice were loaded. 20 µg protein were loaded for spleen, lung, kidney, thymus and 40 µg protein derived from brain homogenates. Equal loading was confirmed by β-actin detection. The intensities of the Gα_i3_ signal were normalized to the relative intensities of the loading control β-actin. Data are represented as mean ± SD and were analyzed using multiple comparison one-way ANOVA followed by post-hoc comparison with Bonferroni’s multiple comparison. Uncropped blots are shown in Supplemental Figure S3.

To study whether the knock-in of GFP into the *Gnai3* locus affects the expression of the Gα_i3_ protein, we employed an isoform-specific antibody directed against the most C-terminal 21 amino acids of Gα_i3_^14^. Gα_i3_ protein levels were quantified in spleen, thymus, lung, kidney, and brain. As expected, organs derived from mice with a global deficiency of *Gnai3* (−/−) lacked the Gα_i3_ protein in all tissues analyzed (Fig. 3A–C, left panels). In contrast, the Gα_i3_ protein was detectable in wild-type, heterozygous and homozygous knock-in mice. Importantly, statistical analysis revealed no significant differences in Gα_i3_ protein expression levels of heterozygous and homozygous knock-in mice compared to wild-type littermates (Fig. 3A–C, right panels). These data suggest that the IRES-GFP insert has negligible effects on Gα_i3_ expression. Consequently, homozygous knock-in mice are suitable for further studies to use GFP expression as a substitute parameter for Gα_i3_ expression.

### GFP can be visualized in thymus, spleen and blood cells using flow cytometry

**Figure 4:**
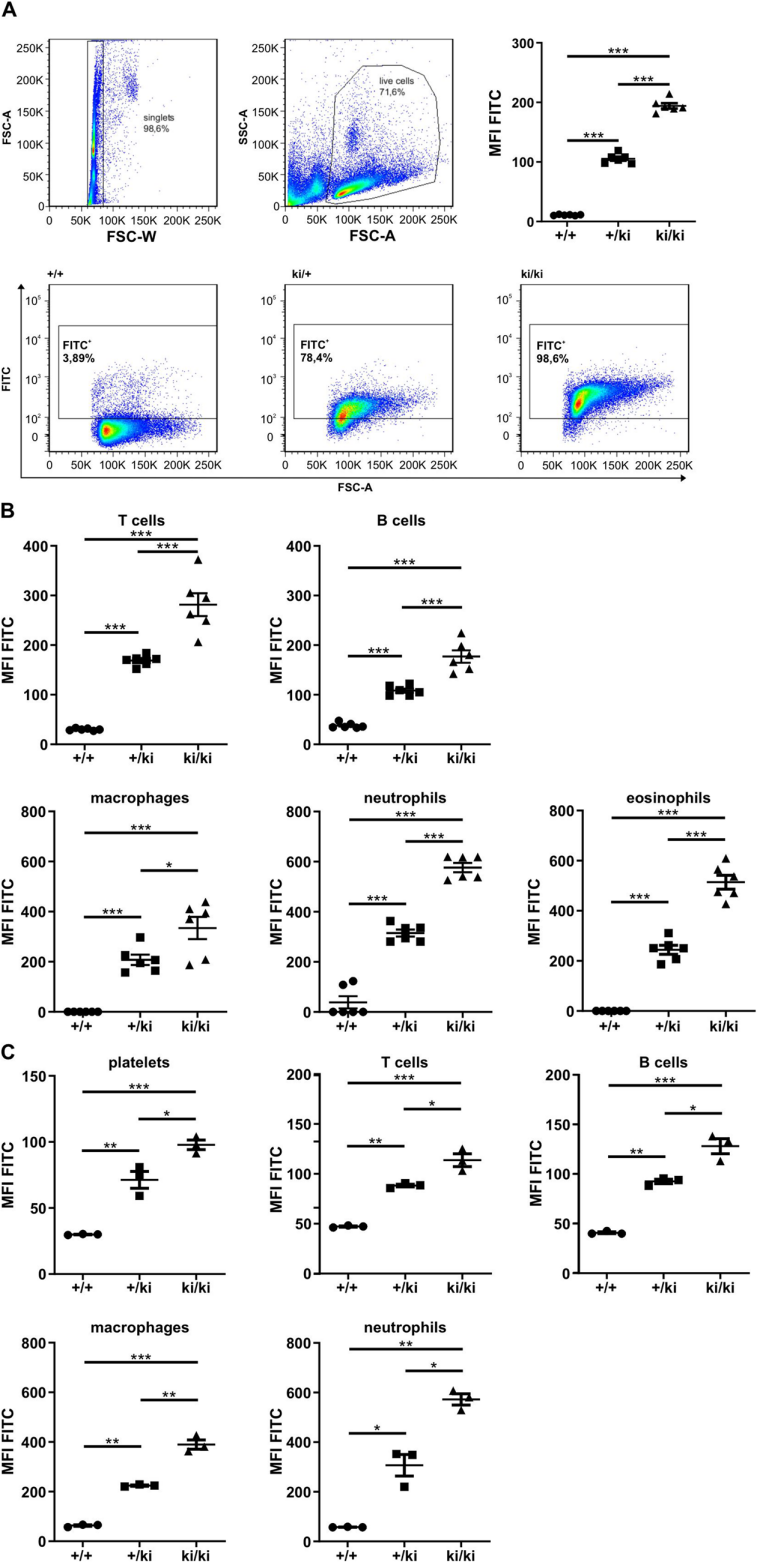
Flow cytometry detection of GFP in *Gnai3*-iresGFP reporter mice. (**A**) Scatter blot analysis of flow cytometry and gating strategy for single cell analysis (upper panel left). Mean fluorescence intensity (MFI) of FITC in thymocytes (upper panel right) and representative FITC detection in wild-type, +/ki and ki/ki thymocytes (lower panel). FITC detection is restricted to +/ki and ki/ki cells. (**B**) MFI of FITC in T cells, B cells, macrophages, neutrophils and eosinophils of spleens of the indicated genotypes. (**C**) MFI of FITC in platelets, T cells, B cells, macrophages and neutrophils in the blood of the indicated genotypes. Whereas no FITC detection was detectable in wild-type cells, the FITC signal was visualized in knock-in mice. The MFI was significantly higher in homozygous knock-in mice (see also Table 1). Data are represented as mean ± SD and were analyzed using multiple comparison one-way ANOVA followed by post-hoc comparison with Bonferroni’s multiple comparison.

Next, we were interested whether GFP fluorescence is directly detectable by flow cytometry in thymocytes, splenic and blood cells (Fig. 4). Of note, the knock-in of GFP did not interfere with immune cell composition of blood and spleen (Supplemental Fig. S1). Whereas all wild-type cell suspensions were negative for GFP fluorescence, GFP expression was evident in thymocytes (Fig. 4A), splenic (Fig. 4B) and blood (Fig. 4C) cell suspensions of *Gnai3* -iresGFP reporter mice carrying either one or two knock-in alleles. Interestingly, the mean fluorescence intensity (MFI) for GFP differed between the cell types and increased significantly from +/ki to ki/ki mice indicating again *Gnai3* transcription from both alleles (Fig. 4; Table 1). Furthermore, GFP fluorescence was verified in all blood and splenic cell populations, e.g., platelets, B cells, T cells, macrophages and granulocytes, derived from *Gnai3* -iresGFP reporter mice suggesting that staining with the cell surface-specific antibodies did not interfere with the GFP detection. Importantly, GFP fluorescence was directly visualized by flow cytometry without the use of primary and/or secondary antibodies. Therefore, our *Gnai3* -iresGFP reporter mouse represents an ideal tool to combine the detection of an intracellular protein in combination with surface markers to conclude on Gα_i3_ expression in different cell populations.

**Table 1:** MFI for indicated cell types of +/ki and ki/ki thymocytes, splenic and blood cells.

Genotype	+/ki	ki/ki
**Cell type**		
Thymocytes	105.5 **±** 4.4	194.2 ± 10.2
**Spleen**		
B cells	108.4 **±** 9.1	177.0 ± 27.8
T cells	168.8 **±** 9.9	281.5 ± 51.7
Macrophages	207.7 **±** 46.5	334.5 ± 99.1
Neutrophils	315.0 **±** 31.4	576.5 ± 41.8
Eosinophils	244.5 **±** 40.1	514.0 ± 62.0
**Blood**		
B cells	92.5 **±** 3.0	128.0 ± 10.0
T cells	88.3 **±** 1.7	113.7±7.6
Macrophages	224.3 **±** 3.1	389.7 ± 24.2
Neutrophils	307.0 **±** 58.0	572.3 ± 28.9
Platelets	73.8 ± 8.1	97.8 ± 4.2

### Distinct GFP expression is observed in the organ of Corti

**Figure 5:**
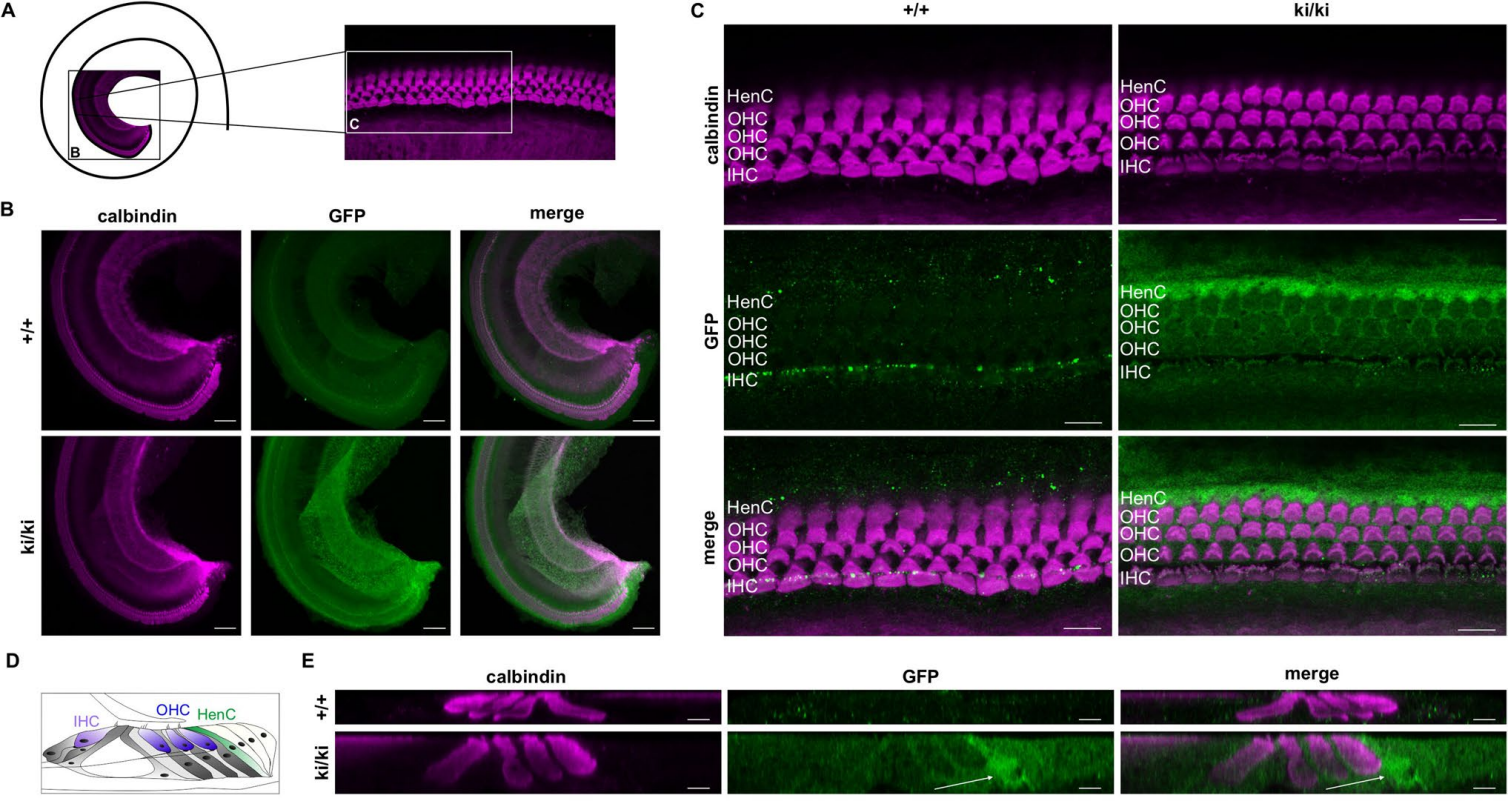
GFP expression in the cochlea of *Gnai3*-iresGFP reporter mice at P8. (**A**) Scheme of a top view onto the apical turn of the cochlea and the sites of low and high magnification views shown in (**B**,**C**). (**B**) Representative low magnification view of a wild-type (top) and a ki/ki (bottom) organ of Corti (P8) stained against calbindin (magenta) and GFP (green). GFP expression is restricted to the ki/ki genotype. Calbindin, used to visualize outer (OHC) and inner hair cells (IHC), is detectable in wild-type and ki/ki organs of Corti. Maximum projection of confocal sections is depicted. (**C**) High magnification views (single optical sections) of OHCs and IHCs immunolabeled for calbindin (magenta) and GFP (green) from wild-type and ki/ki animals (P8). GFP expression was strongest in the first row of Hensen’s cells (HenC). (**D**) Scheme of an orthogonal cross-section through the organ of Corti. (**E**) Virtual orthogonal cross-section through the organ of Corti displaying one IHC, three OHCs and supporting cells of wild-type and ki/ki mice double-labelled for GFP (green) and calbindin (magenta). Arrows indicate HenC. Images of wild-type and ki/ki were acquired and displayed with the same settings. Scale bars (**B**) 20 µm, (**C**) 10 µm and (**E**) 5 µm.

**Figure 6:**
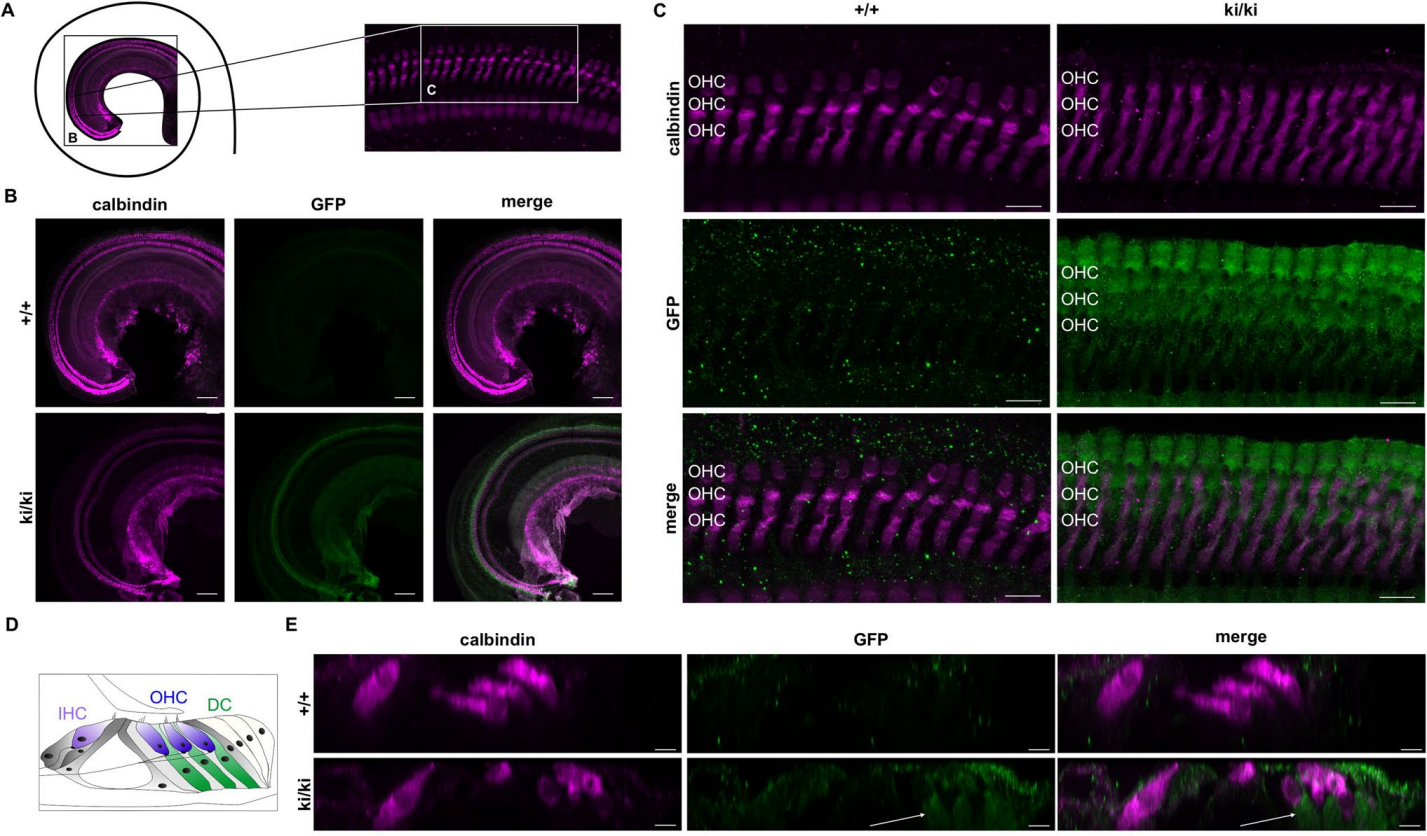
GFP expression in the cochlea of *Gnai3*-iresGFP reporter mice at P21. (**A**) Scheme of a top view onto the apical turn of the cochlea and the sites of low and high magnification views shown in (**B**,**C**). (**B**) Representative low magnification view of a wild-type (top) and a ki/ki (bottom) organ of Corti stained against calbindin (magenta) and GFP (green). GFP expression is restricted to the ki/ki genotype. Calbindin, a marker for IHC and OHC, is detectable in wild-type and ki/ki organs of Corti. Maximum projection of confocal sections is depicted. (**C**) High magnification views of OHCs immunolabeled for calbindin (magenta) and GFP (green) from wild-type and ki/ki animals (P21) as maximum projection of confocal stacks. (**D**) Scheme of an orthogonal cross-section through the organ of Corti. (**E**) Virtual orthogonal section through the organ of Corti, displaying one IHC and three OHCs as well as supporting cells of wild-type and ki/ki mice labelled for GFP (green) and calbindin (magenta). GFP expression was strongest in Deiter’s cells (DC). Arrows indicate DC. Images of wild-type and ki/ki were acquired and displayed with the same settings. Scale bars (**B**) 20 µm, (**C**) 10 µm and (**E**) 5 µm.

Having demonstrated GFP expression by immunoblotting and flow cytometry, we now aimed to verify GFP expression in whole mount samples. As we and others have previously shown that Gα_i3_ proteins have isoform-specific functions in the organ of Corti^15,27–29,32^, we isolated and immunostained cochleae at postnatal day 8 (P8) and P21 from wild-type and homozygous knock-in mice with antibodies against GFP and calbindin, a common marker protein for inner and outer hair cells. As depicted in Figs. 5 and 6, we found GFP expression in the organ of Corti derived from ki/ki animals, whereas wild-type mice lacked specific GFP expression (Figs. 5B,C,E, 6B,C,E). GFP expression was found both in sensory and non-sensory cell types of the organ of Corti. In P8 organs of Corti, high magnification views revealed the strongest GFP expression in supporting cells, namely the first row of Hensen’s cells (Fig. 5C,E). In more mature inner ear tissue (P21), strongest GFP expression appeared in all rows of Deiter’s cells (Fig. 6C,E). Taken together, besides immunoblot analysis, GFP expression can also be visualized with immunostainings. Thus, our *Gnai3* -iresGFP mouse line is suitable to use GFP expression as a putative surrogate to analyze which cells express the Gα_i3_ protein.

## Discussion

Here we show that the *Gnai3*-iresGFP reporter mouse line is a powerful tool to study expression sites of Gα_i3_ proteins with the help of GFP. Within this study, we demonstrate that *Gnai3*-driven GFP expression can be used as an adequate approach to evaluate Gα_i3_ expression pattern. Our conclusion is based on several findings: (1) the IRES-GFP construct does not alter endogenous Gα_i3_ expression, (2) GFP expression is readily detectable using a wide range of biochemical and cell-analysis methods when inserted into the *Gnai3* locus, and (3) GFP expression increases with its number of copies. Thus, GFP fluorescence can be used as an indicator for Gα_i3_ expression.

Both immunoblot analysis using antibodies against GFP and flow cytometry showed that the GFP intensities increased from heterozygous to homozygous knock-in mice. This indicates that both *Gnai3* alleles are transcribed and subsequently translated to generate Gα_i3_ and GFP. Such a gene-dosage effect has already been described for other Gα_i_ isoforms^16,33^.

Interestingly, different MFIs for the respective cell populations were seen in the flow cytometry. Whereas high MFI levels were detected for macrophages, neutrophilic and eosinophilic granulocytes, MFI levels were obviously lower in thymocytes as well as splenic and blood T cells and platelets. As Gα_i3_ and GFP are expressed from a single bicistronic mRNA, GFP fluorescence should correspond to Gα_i3_ expression. Thus, our data suggest a higher expression level of Gα_i3_ in macrophages, neutrophilic and eosinophilic granulocytes compared to the other cell types tested. However, this should be validated by further investigations, as differences in MFIs could be due to differences in the turn-over of GFP and/or Gα_i3_. Moreover, posttranscriptional regulation of translation might be regulated differently in various cell types, giving rise to changes in the Gα_i3_ to GFP ratio.

Several studies have demonstrated isoform-specific functions of Gα_i3_ in the organ of Corti^15,27–29,32^. Gα_i3_ is involved in the directed migration of the kinocilium at the surface of cochlear hair cells during development. In adult Gα_i3_-deficient mice, high frequency hearing was severely affected^15^. Within this study, we confirm the expression of Gα_i3_ indirectly via the detection of GFP in the organ of Corti in our *Gnai3*-iresGFP reporter mice. While previous studies described Gα_i3_ immunostaining close to the cuticular plate of inner and outer hair cells^15,27,28^, we found strongest GFP expression in the first row of Hensen’s cells and in Deiter’s cells. This indicates a high transcriptional rate of the Gα_i3_-IRES-GFP mRNA. Whether this correlates with increased Gα_i3_ protein levels needs further studies. A limiting factor is that GFP expression was not directly detectable in the immunofluorescent staining but only with the help of antigen–antibody complexes. The reasons for this are either weak endogenous GFP expression, the decrease of GFP fluorescence due to PFA fixation, the well-known instability of the GFP protein or a combination of these^34^. Nevertheless, the detection of Gα_i3_ transcripts in cells and organs via endogenous GFP expression simplifies experimental setups as cross-reactivity of Gα_i_ isoform-specific antibodies due to the high sequence homologies is excluded.

Altogether, our study strongly suggests using the *Gnai3*-iresGFP reporter mouse as a valuable tool to analyze Gα_i3_ expression. Studies in this mouse line will potentially indicate more cell types expressing Gα_i3_, as we identified in Hensen’s cells and Deiter’s cells in the inner ear to be sites of Gα_i3_ expression. Moreover, this reporter mouse line will allow studying varying expression levels, e.g., in different developmental stages, responses to external stimuli or pathophysiological conditions.

## Material and methods

### Experimental animals

Global *Gnai3*-deficient mice (−/−) on a C57BL/6N background have been described recently^20^. *Gnai3*-iresGFP mice on a C57BL/6N genetic background were generated in collaboration with Cyagen Biosciences. In Exon 8 of the *Gnai3* gene, the GFP expression cassette, comprising an internal ribosomal entry site (IRES) and the GFP cDNA, was inserted right after the TGA stop codon. GFP protein expression is driven by means of the IRES to achieve independent translation of Gα_i3_ and GFP from the same mRNA^15^. All mice were kept under specific pathogen-free conditions in isolated ventilated cages at the animal facility of the University of Tübingen. Except for the cochlear studies, for which mice at postnatal day 8 (P8) and day 21 (d21) were used, all experiments were performed on 10–14-week-old animals.

The study was carried out in compliance with the ARRIVE guidelines. All experiments were performed according to the EU Animals Scientific Procedures Act and the German law for the welfare of animals. All procedures were approved by the authorities of the state of Baden-Württemberg, namely the Regierungspräsidium Tübingen (permission number PH4/14 and PH5/19M).

### Genotyping of mice

Ear punches were mixed with DirectPCR lysis reagent (Viagen, Los Angeles, CA, USA) supplemented with proteinase K (GeneOn, Ludwigshafen, Germany) and incubated at 55 °C overnight. Finally, proteinase K was inactivated at 85 °C. The following primers were used for amplification: primer 1: 5′-AGTCAAATGGCTCTCCTCAAGCGTA-3′; primer 2: 5′-TACCCCGCCCCCAGTGGTAA-3′; primer 3: 5′-TAACTTAGCTGGGTG CAGGCA-3′. The knock-in fragment (size 1200 bp) was amplified with primers 1 and 2. The wild-type product (300 bp) was detected using primers 2 and 3. PCR products were analyzed with the QIAxcel system (Qiagen, Venlo, Netherlands).

### Immunoblot analysis

Dissected organs were homogenized in RIPA buffer (65 mM Tris, 150 mM NaCl, 1 mM EDTA, 1% NP40, 1 µM DTT, 1 µM protease inhibitor (cOmplete Mini, Roche, Mannheim, Germany) using an ULTRA-TURRAX (IKA, Staufen, Germany) to generate total protein lysates for subsequent immunoblot analyses. The proteins were separated by their molecular weight using 12% SDS gels containing 6 M urea to achieve proper electrophoretic separation of Gα_i_ isoforms, and subsequently transferred onto polyvinylidene difluoride membranes (Immobilon^®^-P, Merck, Darmstadt, Germany) by semi-dry blotting. Unspecific antibody binding was blocked with either 5% BSA or 5% milk-TBST (tris-buffered saline-Tween 20). Primary antibodies used for immunoblotting were anti-GFP (abcam, Cambridge, UK), anti-β-actin (abcam, Cambridge, UK) and anti-Gα_i3_^8,16^. As secondary antibodies HRP-conjugated anti-rabbit IgG antibodies (1:2000; Cell Signaling, Danvers, USA) were used. Protein-antibody complexes were visualized using Amersham™ ECL™ Prime (GE Healthcare, Chicago, USA) and a VersaDoc 4000 MP imaging system (Bio-Rad, Hercules, USA). The protein levels were quantified using densitometric analysis software (Image Lab, Bio-Rad, Hercules, USA) and normalized to the β-actin levels of the same samples.

### Immunostaining

Immunostaining of inner ears was performed essentially as described in Strenzke et al.^35^ with the following modifications: cochleae were fixed in 4% PFA for 1 h. Primary antibodies were incubated for 72 h at 4 °C. After the first antibody, cochleae were washed once with wash buffer (20 mM phosphate buffer pH 7.4, 0.3% Triton X-100, 450 mM NaCl) and twice with PBS (Sigma Aldrich Merck, Darmstadt, Germany), each wash step for 10–15 min. After the secondary antibody incubation (overnight, 4 °C), cochleae were washed 3 × 10 min with PBS. Tissue was embedded in FluoSafe™ (Calbiochem/Merck, Darmstadt, Germay). The following antibodies were used: chicken IgY anti GFP (#ab13970, Abcam, 1:400^36^), rabbit anti calbindin D28k (#CB-38a, Swant, 1:400), Donkey anti chicken IgY 488 (#703-546-155, Jackson ImmunoResearch, 1:200) and Donkey anti rabbit Alexa Fluor 546 (# A10040, lifetechnologies, 1:200).

### Imaging

Confocal images were acquired on a Leica SP8 microscope with 20× oil immersion objective (0.75× zoom) for low magnification images and a 63× oil immersion objective for high magnification views and 488 nm and 532 nm excitation lasers. Z-stacks were acquired with step size of 0.3 µm. Imaging files were loaded in *ImageJ* and single optical sections or maximum intensity projections were generated. Virtual orthogonal sections through the z-stacks were generated with the “Orthogonal Views” tool in *ImageJ*.

### Preparation of cell suspensions from spleen, thymus and blood

Thymus and spleen were mashed through a 70 µm filter (cell strainer; BD) to get single cell suspensions. Cell suspensions derived from spleens and citrated blood were additionally incubated in erythrocyte lysis buffer (155 mM NH_4_Cl, 10 mM KHCO_3_ and 0.1 mM EDTA) for 3 min to avoid interference of erythrocytes with flow cytometry staining. After centrifugation, the pellets were resuspended in PBS and the cells were counted in a Neubauer haemocytometer. For flow cytometry 1 × 10^6^ cells were analyzed.

### Flow cytometry

Whereas thymus cells were directly subjected to flow cytometry, blood and splenic cells were subsequently stained with cell-type specific surface markers. After Fc blocking with CD16/CD32, splenic cells were incubated with the following antibodies for 15 min on ice: anti-mouse F4/80-Pacific Blue, anti-mouse Siglec F-PE, anti-mouse CD45R/B220-PerCP/Cy5.5, anti-mouse CD3ε-PE-Cy 7, anti-mouse Ly6G (Gr-1)-APC, anti-mouse CD11b-BrilliantViolet 510, and anti-mouse CD11c-APC-Cy 7. Blood cells were stained with anti-mouse CD3ε-PE-Cy 7, anti-mouse CD19-V450, anti-mouse CD11b-PerCPCy5.5, anti-mouse F4/80-PE and anti-mouse Ly6G-APC for detection of macrophages and neutrophils and with anti-mouse CD3ε-PE-Cy 7, anti-mouse CD19-V450, anti-mouse CD11b-PerCPCy5.5 and anti-mouse CD41-APC for detection of platelets, T and B cells. All antibodies were purchased from BD bioscience (Becton Dickinson, Franklin Lakes, USA) or BioLegend (San Diego, USA). Single cell suspensions were analyzed with a BD FACSCantoTM II machine (Becton Dickinson, Franklin Lakes, USA). The obtained data were evaluated using FlowJo software (Becton Dickinson, Franklin Lakes, USA).

### Gating strategy

For flow cytometry of lymphocytes living cells were gated using the forward (FSC-A) and sideward (SSC-A) scatter. In splenic cell suspensions, B cells were defined as B220^+^ and CD3ε^−^, whereas T cells were defined as B220^−^ and CD3ε^+^. Macrophages were gated as CD3ε^−^ CD19^−^ CD11b^+^ CD11c^−^ F4/80^+^ cells, eosinophils as CD3ε^−^ CD19^−^ CD11b^+^ CD11c^−^ Siglec-F^+^ cells and neutrophils as CD3ε^−^ CD19^−^ CD11b^+^ CD11c^−^ Siglec-F^−^ cells expressing Ly6G.

In the blood, B (CD3e− CD19+) and T cells (CD3e+ CD19−) were gated in a CD3ε and CD19 blot and defined. Next, CD11b^+^ cells were gated using a F4/80 and Ly6G blot, with neutrophils defined as positive for both markers and macrophages positive for F4/80 only. Platelets were stained separately and defined as B220^−^ CD3^−^ CD11b^−^ CD41^+^ cells.

### Statistical analysis

All data are presented as mean ± SD. Statistical analysis was performed using two-way analysis of variance (ANOVA) with the post hoc Bonferroni test for multiple comparison. All calculations were performed using GraphPad Prism 9 (GraphPad Software, La Jolla CA, USA). A value of *P* ≤ 0.05 was considered statistically significant.

## Supplementary Information

Supplementary Information 1.

Supplementary Information 2.

Supplementary Information 3.

Supplementary Information 4.

## Abbreviations

DC: Deiter’s cell
DTA: Diphtheria Toxin A
GFP: Green fluorescent protein
GPCR: G protein-coupled receptor
HenC: Hensen’s cell
IHC: Inner hair cell
IRES: Internal ribosomal entry site
ki: Knock-in allele
MFI: Mean fluorescence intensity
OHC: Outer hair cell
SD: Standard deviation
SDS-PAGE: Sodium dodecyl sulfate polyacrylamide gel electrophoresis
+: Wild-type allele
