# Supplementary figures and images for: Analyses of Gnai3-iresGFP reporter mice reveal unknown Gαi3 expression sites
Source: Sci Rep. 2021 Jul 12;11:14271. doi: 10.1038/s41598-021-93591-0 (PMC8275620; doi:10.1038/s41598-021-93591-0)

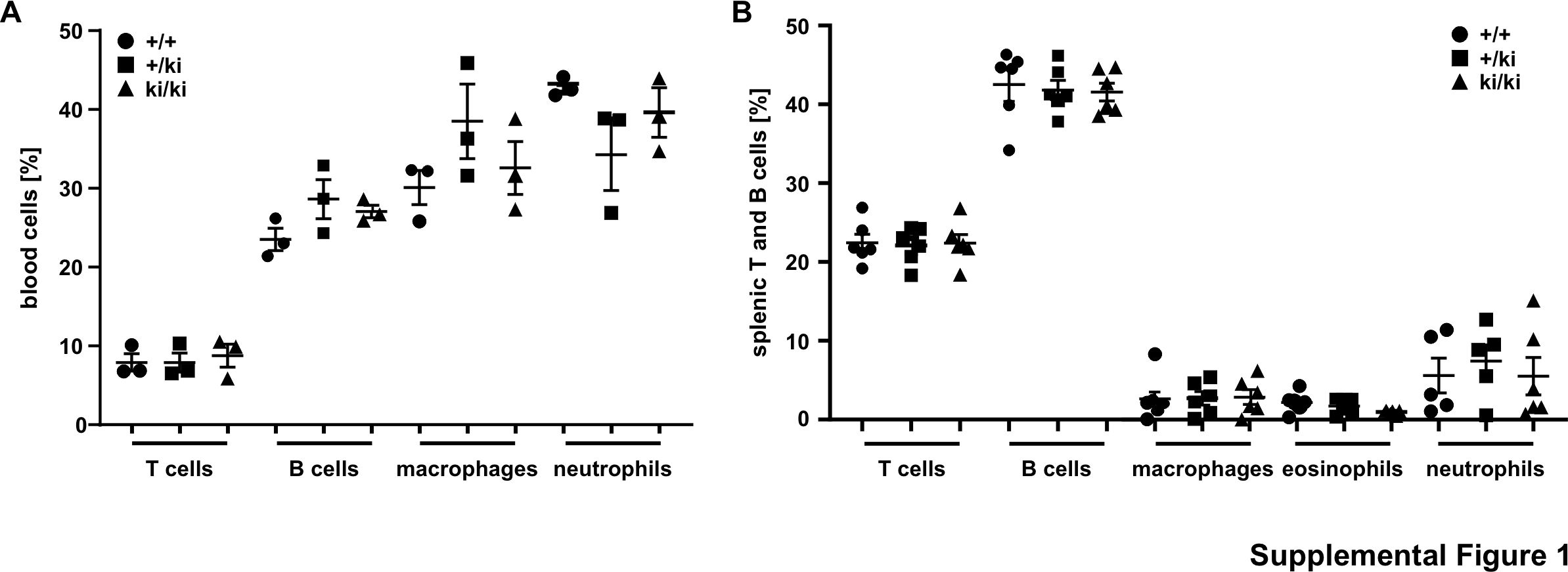

Supplement: Supplementary file 1 — Supplementary Information 1. [file 41598_2021_93591_MOESM1_ESM.jpg]

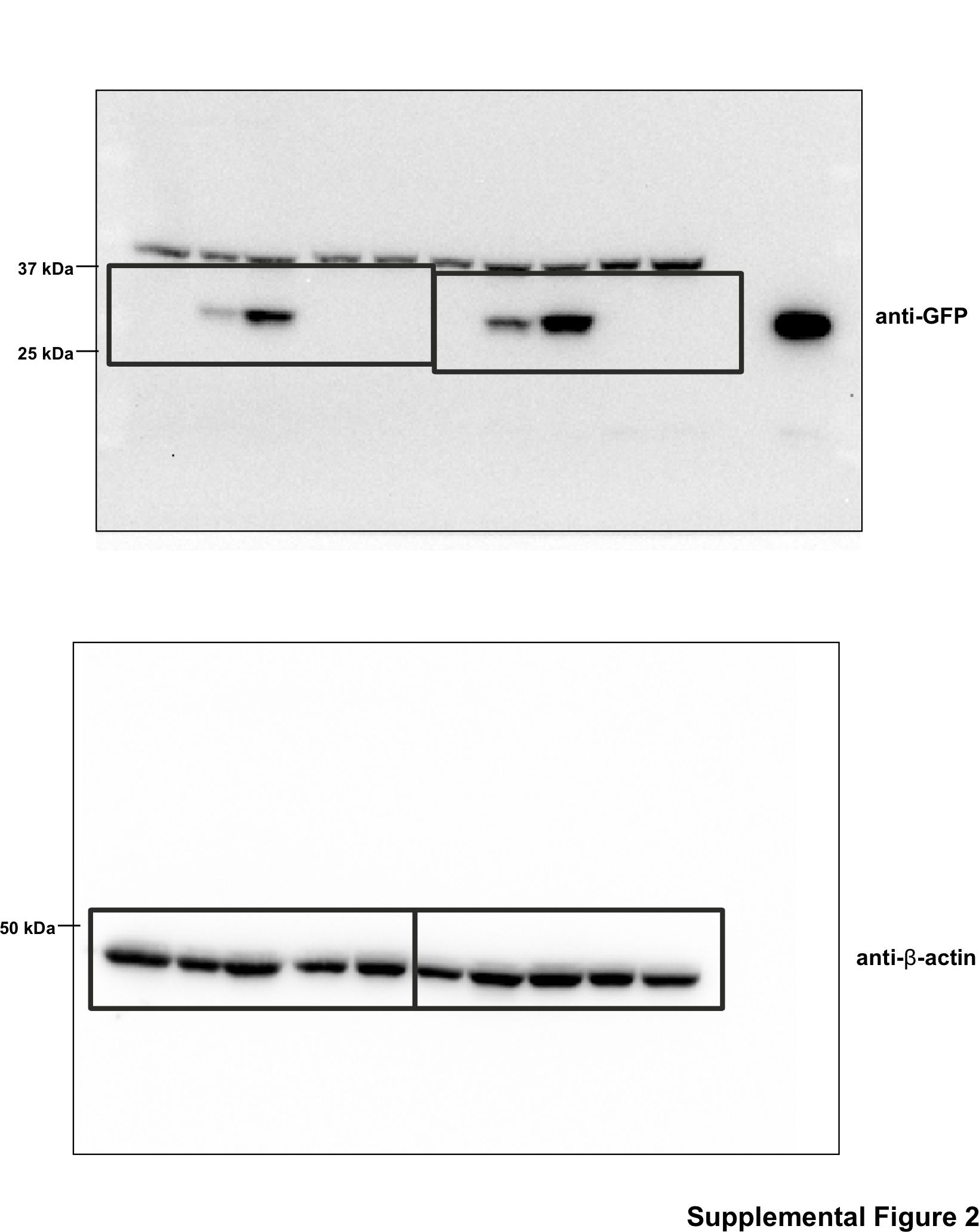

Supplement: Supplementary file 2 — Supplementary Information 2. [file 41598_2021_93591_MOESM2_ESM.jpg]

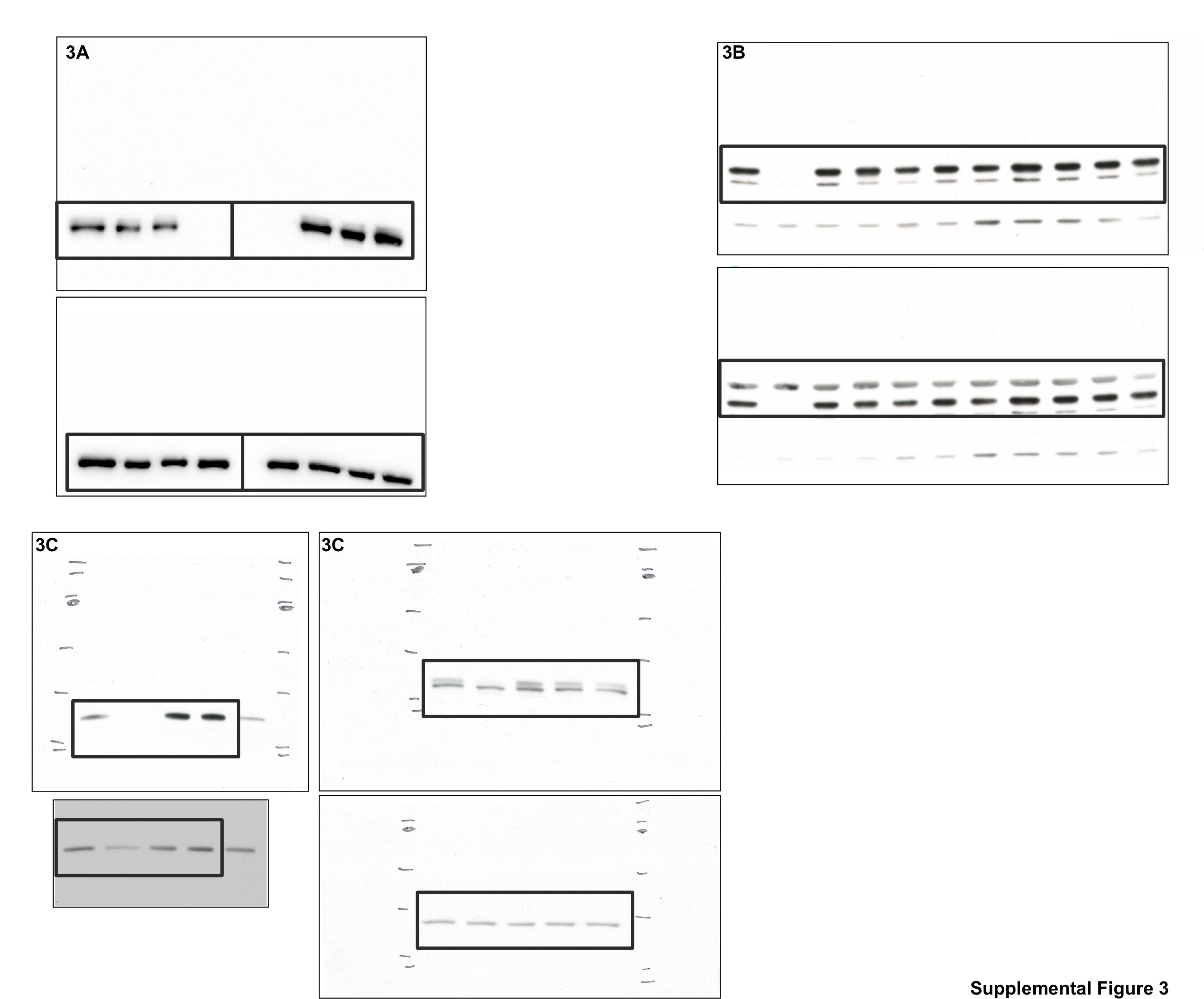

Supplement: Supplementary file 3 — Supplementary Information 3. [file 41598_2021_93591_MOESM3_ESM.jpg]
