# Supplementary material for: Analyses of Gnai3-iresGFP reporter mice reveal unknown Gαi3 expression sites
Source: Sci Rep. 2021 Jul 12;11:14271. doi: 10.1038/s41598-021-93591-0 (PMC8275620; doi:10.1038/s41598-021-93591-0)
Supplement: Supplementary file 4 — Supplementary Information 4. [file 41598_2021_93591_MOESM4_ESM.pdf]

Analyses of *Gnai3*-iresGFP reporter mice reveal unknown $G\alpha_{i3}$ expression sites

Veronika Leiss[1], Ellen Reisinger[2], Annika Speidel[1], Sandra Beer-Hammer[1]*, Bernd Nürnberg[1]

[1] Department of Pharmacology, Experimental Therapy and Toxicology, Institute of Experimental and Clinical Pharmacology and Pharmacogenomics, and ICePhA Mouse Clinic, University of Tübingen, Wilhelmstraße 56, D-72074 Tübingen

[2] Department of Otolaryngology - Head & Neck Surgery, Gene Therapy for Hearing Impairment Group, University of Tübingen, Medical Center, Elfriede-Aulhorn-Straße 5, 72076 Tübingen

*Corresponding author:

Prof. Dr. Sandra Beer-Hammer
Institute of Experimental and Clinical Pharmacology and Pharmacogenomics
University Tübingen
Wilhelmstraße 56
D-72074 Tuebingen
phone: 49-7071-29-74594
fax: 49-7071-29-4942
sandra.beer-hammer@uni-tuebingen.de

Supplemental Fig. 1: Cell composition in blood and spleen

Percentage of T cells, B cells, macrophages and neutrophils in A) blood and B) spleen of wild type, heterozygous and homozygous *Gnai3*-iresGFP reporter mice.

Supplemental Fig. 2: Uncropped immunoblot images shown in Figure 2.

Boxes indicate depicted images in Fig. 2

Supplemental Figure 3: Uncropped immunoblot images shown in Figure 3.

Boxes indicate depicted images and are numbered correspondingly to Fig. 3.
